# Characteristics of the complete chloroplast genome of *Halophila beccarii*

**DOI:** 10.1080/23802359.2020.1715879

**Published:** 2020-01-22

**Authors:** Shuo Yu, Xiaojuan Li, Kai Jiang, Xuyang Chen, Xiangping Wang

**Affiliations:** a Ministry of Natural Resources, Fourth Institute of Oceanography, Beihai, China;; b Key Laboratory of Plant Resources Conservation and Sustainable Utilization, South China Botanical Garden, Chinese Academy of Sciences, Guangzhou, China;; c Shanghai Chenshan Plant Science Research Center, Chinese Academy of Sciences, Chenshan Botanical Garden, Shanghai, China;; d School of Ecological and Environmental Sciences, Shanghai Key Lab of Urban Ecological Processes and Eco-Restoration, East China Normal University, Shanghai, China

**Keywords:** *Halophila beccarii*, Illumina sequencing, plastid genome, phylogenetic relationship

## Abstract

*Halophila beccarii* has been listed as a vulnerable species in ICNU. In this study, the complete plastid genome sequence of *H. beccarii* was successfully sequenced by the technology of Illumina. The whole plastid genome length was 168,585 bp and contained a typical quadripartite structure including one large single-copy (LSC) region (80,881 bp), one small single-copy (SSC) region (4,730bp) and a pair of inverted repeats (IR) regions (41,487bp). The GC content of this genome was 38.5%. The whole genome contained 132 genes including 88 protein-coding genes, 36 tRNA genes, and 8 rRNA genes. The phylogenetic analysis indicated that *H. beccarii* and *Thalassia hemprichii* formed a distinct clade.

The genus *Halophila* (Hydrocharitaceae) has the greatest diversity of seagrass species, and distributed along the coastlines worldwide (Short et al. 2007). The taxonomy of this genus has long been confused due to its high plasticity in morphology (Kuo et al. 2006). So far, 17 *Halophila* species have been reported (Short et al. 2011). *Halophila beccarii*, a small seagrass species, is characterized by distinct erect lateral shoots with a pseudo-whorl of 4–10 sheathing petiolate tiny leaves (den Hartog and Kuo 2006). This species exhibits both clonal and sexual reproduction, and mainly occurs along the coasts of south-east Asia. However, its distribution area has experienced a rapid decline due to anthropogenic disturbances (Jiang et al. 2014). Now this species has been listed as a vulnerable species on the IUCN Red List of threatened seagrass species (Short et al. 2011). In this study, we sequenced the complete plastid genome of *H. beccarii* using next-generation technology. Information about this genome will be very useful in taxonomy and phylogenetic studies for *Halophia*.

Fresh plant of *H. beccarii* was collected from Chengmai, Hainan Province, China (19.93°N, 109.98°E), and the specimen was stored at Fourth Institute of Oceanography Herbarium (CM201908-1). After cleaning the attached epiphytes with fresh water, and then the genomic DNA was extracted using Plant Genomic DNA kit (Tiangen, Beijing, China), and sequenced using the Illumina Novaseq platform. Low-quality reads and adapters were removed by the FastQC software (Andrews 2010). *De novo* genome assembly was conducted by SPAdes v3.9 (Bankevich et al. 2012). The complete plastid genome was annotated using GeSeq (Tillich et al. 2017) with default sets. The annotations of tRNA genes were performed by ARAGORN (Laslett and Canback 2004). The complete plastid genome of *H. beccarii* was submitted to GenBank database (Accession Number: MN736637).

In *H. beccarii*, the complete plastid genome is 168,585 bp in length with a typical structure including one large single-copy region (80,881 bp), one small single-copy region (4730 bp) and a pair of inverted repeats (IRs) (41,487 bp). The guanine-cytosine (GC)-content is 38.5%. A total of 132 genes in this genome consisted of 88 protein-coding genes, 36 tRNA genes, and 8 rRNA genes. There are 29 duplicated genes in the IR regions including 18 protein-coding genes (infA, ndhF, rpl2, rpl14, rpl16, rpl22, rpl23, rpl32, rpl36, rpoA, rps3, rps7, rps8, rps11, rps12, rps19, ycf15 and ycf2), 7 tRNA genes (trnA-UGC, trnH-GUG, trnI-CAU, trnI-GAU, trnL-CAA, trnR-CCG and trnV-GAC), and 4 rRNA genes (rrn16, rrn23, rrn4.5 and rrn5).

To clarify the phylogenetic position of *H. beccarii*, we then downloaded 24 completed plastid genomes from GenBank database. The phylogenetic tree was reconstructed with MEGA6 software (Tamura et al. 2013) using the maximum likelihood (ML) method (Figure 1). Bootstrap values were calculated using 1000 replicates. The phylogenetic analysis indicated that *H. beccarii* and *Thalassia hemprichii* formed a distinct clade.

**Figure 1.**
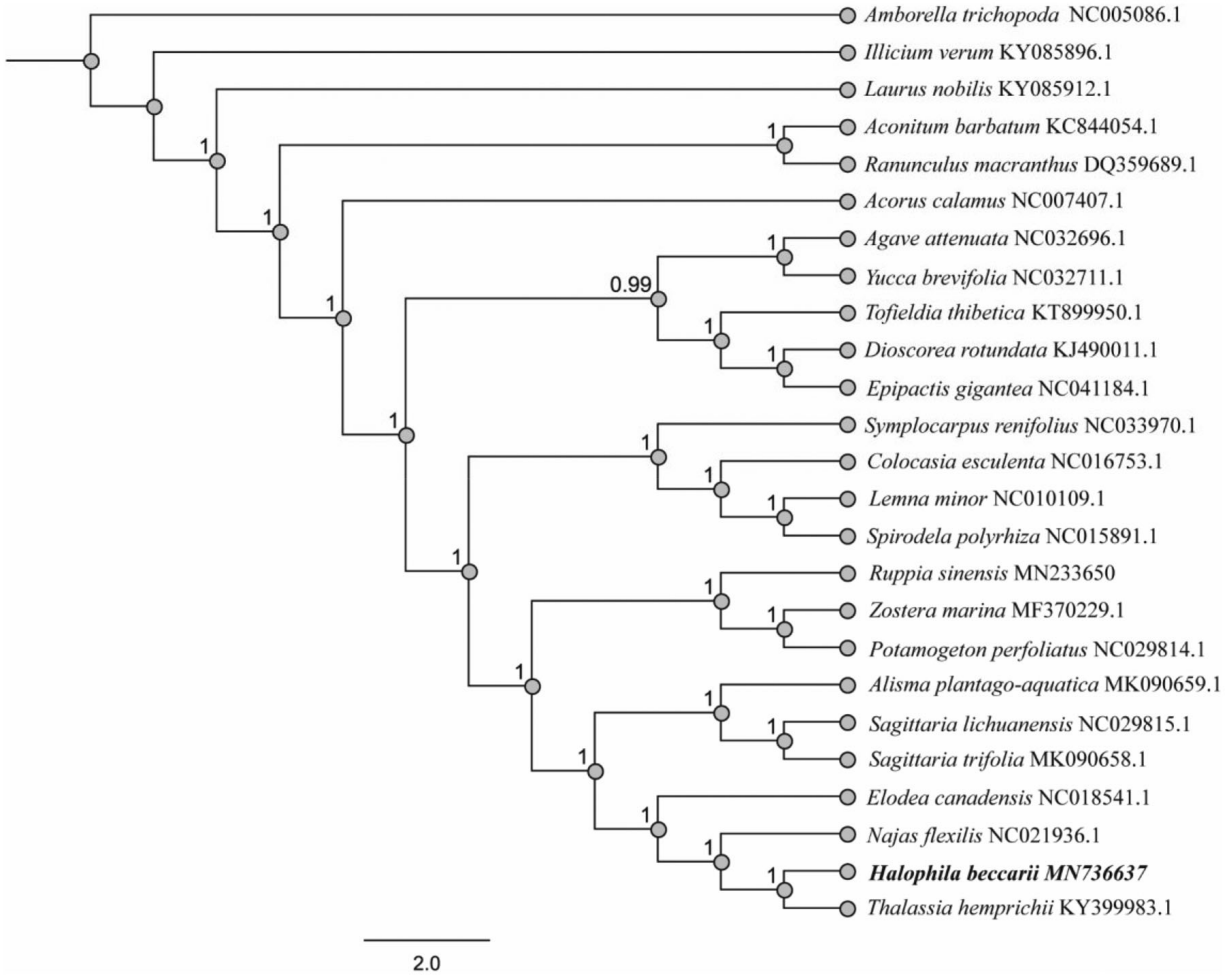
Phylogenetic relationship of 25 species based on the plastid genome sequences with maximum likelihood (ML) analysis.

## Geolocation information

Chengmai, Hainan Province, China (19.93°N, 109.98°).

## References

[CIT0001] Andrews S. 2010. FastQC: a quality control tool for high throughput sequence data. Cambridge, UK: Babraham Institute.

[CIT0002] Bankevich A, Nurk S, Antipov D, Gurevich AA, Dvorkin M, Kulikov AS, Lesin VM, Nikolenko SI, Pham S, Prjibelski AD, et al. 2012. SPAdes: a new genome assembly algorithm and its applications to single-cell sequencing. J Comput Biol. 19(5):455–477. 22506599 10.1089/cmb.2012.0021 PMC3342519

[CIT0003] den Hartog C, Kuo J. 2006. Taxonomy and biogeography of seagrasses. In: Larkum AWD, Orth RJ, Duarte CM, editors Seagrass: biology, ecology and conservation. Dordrecht, The Netherlands: Springer; p. 1–23.

[CIT0004] Jiang K, Xu NN, Tsang PKE, Chen XY. 2014. Genetic variation in populations of the threatened seagrass *Halophila beccarii* (Hydrocharitaceae). Biochem Syst Ecol. 53:29–35.

[CIT0005] Kuo J, Kanamoto Z, Iizumi H, Mukai H. 2006. Seagrasses of the Genus *Halophila* Thouars (Hydrocharitaceae) from Japan. Acta Phytotax Geobot. 57:129–154.

[CIT0006] Laslett D, Canback B. 2004. ARAGORN, a program to detect tRNA genes and tmRNA genes in nucleotide sequences. Nucleic Acids Res. 32(1):11–16. 14704338 10.1093/nar/gkh152 PMC373265

[CIT0007] Short F, Carruthers T, Dennison W, Waycott M. 2007. Global seagrass distribution and diversity: a bioregional model. J Exp Mar Biol Ecol. 350(1–2):3–20.

[CIT0008] Short FT, Polidoro B, Livingstone SR, Carpenter KE, Bandeira S, Bujang JS, Calumpong HP, Carruthers TJB, Coles RG, Dennison WC, et al. 2011. Extinction risk assessment of the world’s seagrass species. Biol Conserv. 144(7):1961–1971.

[CIT0009] Tamura K, Stecher G, Peterson D, Filipski A, Kumar S. 2013. MEGA6: molecular evolutionary genetics analysis version 6.0. Mol Biol Evol. 30(12):2725–2729. 24132122 10.1093/molbev/mst197 PMC3840312

[CIT0010] Tillich M, Lehwark P, Pellizzer T, Ulbricht-Jones ES, Fischer A, Bock R, Greiner S. 2017. GeSeq - versatile and accurate annotation of organelle genomes. Nucleic Acids Res. 45(W1):W6–W11. 28486635 10.1093/nar/gkx391 PMC5570176

